# Enterococcus faecalis Enhances Expression and Activity of the Enterohemorrhagic Escherichia coli Type III Secretion System

**DOI:** 10.1128/mBio.02547-19

**Published:** 2019-11-19

**Authors:** Elizabeth A. Cameron, Vanessa Sperandio, Gary M. Dunny

**Affiliations:** a Department of Microbiology, University of Texas Southwestern Medical Center, Dallas, Texas, USA; b Department of Biochemistry, University of Texas Southwestern Medical Center, Dallas, Texas, USA; c Department of Microbiology and Immunology, University of Minnesota, Minneapolis, Minnesota, USA; Washington University School of Medicine

**Keywords:** commensal pathogen interaction, gut microbiome, virulence regulation, EHEC, bacterial communication

## Abstract

The gut microbiota can significantly impact invading pathogens and the disease they cause; however, many of the mechanisms that dictate commensal-pathogen interactions remain unclear. Enterohemorrhagic Escherichia coli (EHEC) is a potentially lethal human intestinal pathogen that uses microbiota-derived molecules as cues to efficiently regulate virulence factor expression. Here, we investigate the interaction between EHEC and Enterococcus faecalis, a common human gut commensal, and show that E. faecalis affects both expression and activity of the EHEC type III secretion system (T3SS) via two distinct mechanisms. First, in the presence of E. faecalis there is increased transcription of genes encoding the EHEC T3SS. This leads to increased effector translocation and ultimately greater numbers of pedestals formed on host cells. The same effect was observed with several strains of enterococci, suggesting that it is a general characteristic of this group. In a mechanism separate from E. faecalis*-*induced transcription of the T3SS, we report that an E. faecalis-secreted protease, GelE, cleaves a critical structural component of the EHEC T3SS, EspB. Our data suggest that this cleavage actually increases effector translocation by the T3SS, supporting a model where EspB proteolysis promotes maximum T3SS activity. Finally, we report that treatment of EHEC with E. faecalis-conditioned cell-free medium is insufficient to induce increased T3SS expression, suggesting that this effect relies on cell contact between E. faecalis and EHEC. This work demonstrates a complex interaction between a human commensal and pathogen that impacts both expression and function of a critical virulence factor.

## Abstract (precis)

This work reveals a complex and multifaceted interaction between a human gut commensal, Enterococcus faecalis, and a pathogen, enterohemorrhagic E. coli. We demonstrate that E. faecalis enhances expression of the enterohemorrhagic E. coli type III secretion system and that this effect likely depends on cell contact between the commensal and the pathogen. Additionally, the GelE protease secreted by E. faecalis cleaves a critical structural component of the EHEC type III secretion system. In agreement with previous studies, we find that this cleavage actually increases effector protein delivery into host cells by the secretion system. This work demonstrates that commensal bacteria can significantly shape expression and activity of pathogen virulence factors, which may ultimately shape the progression of disease.

## INTRODUCTION

Invading intestinal pathogens encounter a dense microbial community containing hundreds of different bacterial species, each with its own biochemical repertoire. The interactions between pathogens and members of the gut microbiota are complex, and both antagonistic and mutualistic relationships have been previously described (1). The state of the gut microbiota can profoundly affect host susceptibility to infection (2–5) and the severity of disease that develops (6–8), and thus understanding the interactions between commensals and pathogens is critical to understanding the progression of infectious intestinal disease.

Enterohemorrhagic Escherichia coli (EHEC) is a human foodborne pathogen that can cause severe bloody diarrhea, which can progress to the potentially lethal condition hemolytic uremic syndrome. Compared with other intestinal pathogens, EHEC has a remarkably low infectious dose, with 10 to 100 organisms being sufficient to cause disease (9), underscoring the observation that EHEC has evolved extremely efficient mechanisms for competing with resident commensals and expanding in the gut, despite being vastly outnumbered. One strategy EHEC employs toward this goal is using microbiota-derived molecules both as nutrients and as signals to regulate its virulence genes (10).

**FIG 1:**
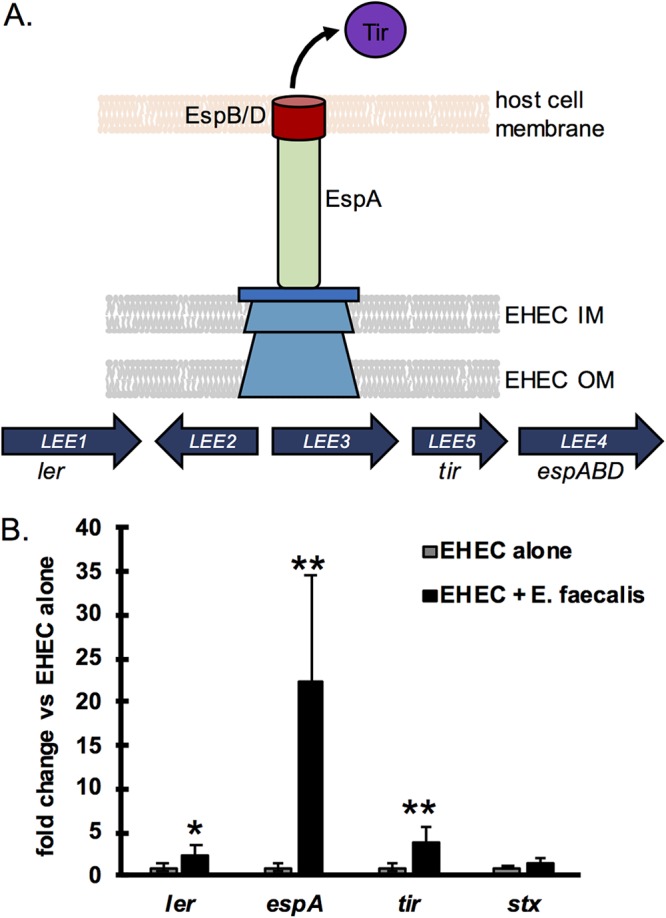
E. faecalis enhances EHEC LEE expression. (A) A schematic of the EHEC locus of enterocyte effacement (LEE) pathogenicity island and the EHEC type III secretion system (T3SS) which it encodes. The LEE is composed of five operons, *LEE1-LEE5*, containing all the genes necessary for the type III secretion system apparatus. Genes of interest discussed in this work are noted below their respective LEE operons. The EHEC T3SS translocon is composed of EspA, which forms the filament, and EspB and EspD that form a pore in the target cell membrane through which effector proteins, such as Tir, are injected into host cells. IM, inner membrane; OM, outer membrane. (B) EHEC was grown alone or in coculture with E. faecalis strain V583 in 1 g/liter glucose with DMEM, and the transcription of the indicated EHEC virulence genes was quantified via qPCR. Virulence genes were normalized to *rpoA* levels, and results are expressed as fold change compared to the levels with EHEC alone. The average and standard deviation of four replicate samples are plotted, and a two-tailed *t* test was performed to determine significant differences compared to results with EHEC alone. *, *P* < 0.05; **, *P* < 0.01.

EHEC expresses two major virulence factors: Shiga toxin, which is responsible for the kidney damage associated with EHEC infection, and a type III secretion system (T3SS), which is required for colonization of the intestine and responsible for much of the intestinal damage (11). The T3SS injects effector proteins into intestinal epithelial cells, some of which co-opt host signaling pathways to promote host cytoskeleton rearrangement and formation of an actin pedestal beneath the attached bacterium. This process causes local destruction of the intestinal microvilli, known as an attaching and effacing (A/E) lesion (12). The EHEC T3SS is encoded on a pathogenicity island (Fig. 1A) known as the locus of enterocyte effacement (LEE), which is tightly regulated via a complex signaling cascade that incorporates signals from the host and microbiota (13). For example, EHEC senses fucose residues liberated from the mucosal layer by the microbiota and decreases expression of the LEE in response (14), while the microbiota-produced metabolite succinate increases LEE expression by EHEC (6). It is clear that there is a complex relationship between EHEC and the microbiota and that members of this community may influence the progression of EHEC-caused disease. However, an understanding of the mechanistic basis of interactions between EHEC and commensal bacteria is still lacking.

In this work we explore how different commensal species affect expression of the EHEC LEE, and we explore in detail the interactions between EHEC and Enterococcus faecalis. Although it is often studied in its role as an opportunistic pathogen, E. faecalis is a common member of the normal gut microbiota in healthy individuals (15). Unlike much of the microbiota, E. faecalis is not a strict anaerobe and is able to directly colonize the intestinal epithelium (16), where EHEC attaches and deploys its T3SS, making the interaction between these two species of particular interest.

We find that E. faecalis both modulates LEE gene expression and proteolytically cleaves a structural component of the T3SS. This presents an intriguing model by which commensals can not only impact gene expression but also directly process pathogen virulence factors. By elucidating the molecular mechanisms that govern the complex interactions between commensals and pathogens, we can more fully understand how the microbiota shapes intestinal disease.

## RESULTS

### Enterococci enhance transcription and activity of the EHEC T3SS.

In the human intestinal tract, pathogens encounter hundreds of different bacterial species which can impact both their colonization and virulence gene expression (10). To investigate how different commensals impact EHEC virulence gene expression, we selected three phylogenetically diverse commensal bacteria for a series of coculture experiments. EHEC was grown with one or more of the species Bacteroides thetaiotaomicron, Enterococcus faecalis strain V583, and E. coli strain HS, and virulence gene expression was measured via quantitative PCR (qPCR) (Fig. 1B; see also Fig. S1A and B in the supplemental material). In agreement with previous reports (6, 17), both B. thetaiotaomicron and E. faecalis enhanced expression of EHEC LEE genes (Fig. 1B and Fig. S1A). When both species were present, there was an additive effect, and LEE expression was higher than that with either species alone. Interestingly, E. coli HS had no effect on the transcription of EHEC virulence genes, but the presence of HS did not inhibit the enhancing effect of either B. thetaiotaomicron or E. faecalis (Fig. S1B).

10.1128/mBio.02547-19.1 FIG S1 Commensal bacteria alter EHEC LEE transcription. (A) EHEC was grown alone or in coculture with E. faecalis strain V583 and/or Bacteroides thetaiotaomicron in 1 g/liter glucose DMEM, and the transcription of the indicated EHEC virulence genes was quantified via qPCR. Virulence gene expression was normalized to *rpoA* transcript levels and expressed as fold change compared to EHEC alone. The average and standard deviation of 4 replicate samples are plotted. (B) EHEC was grown alone or with one or more of the commensal strains: E. faecalis, B. thetaiotaomicron, or E. coli strain HS. To facilitate qPCR normalization in coculture with HS (whose standard housekeeping genes are nearly identical in sequence), a strain of EHEC containing an engineered housekeeping gene was used (see Materials and Methods), and virulence genes were normalized to levels of this engineered housekeeping gene. The average and standard deviation of 4 replicate samples are plotted. Download FIG S1, TIF file, 2.4 MB. Copyright © 2019 Cameron et al. 2019 Cameron et al. This content is distributed under the terms of the Creative Commons Attribution 4.0 International license.

**FIG 2:**
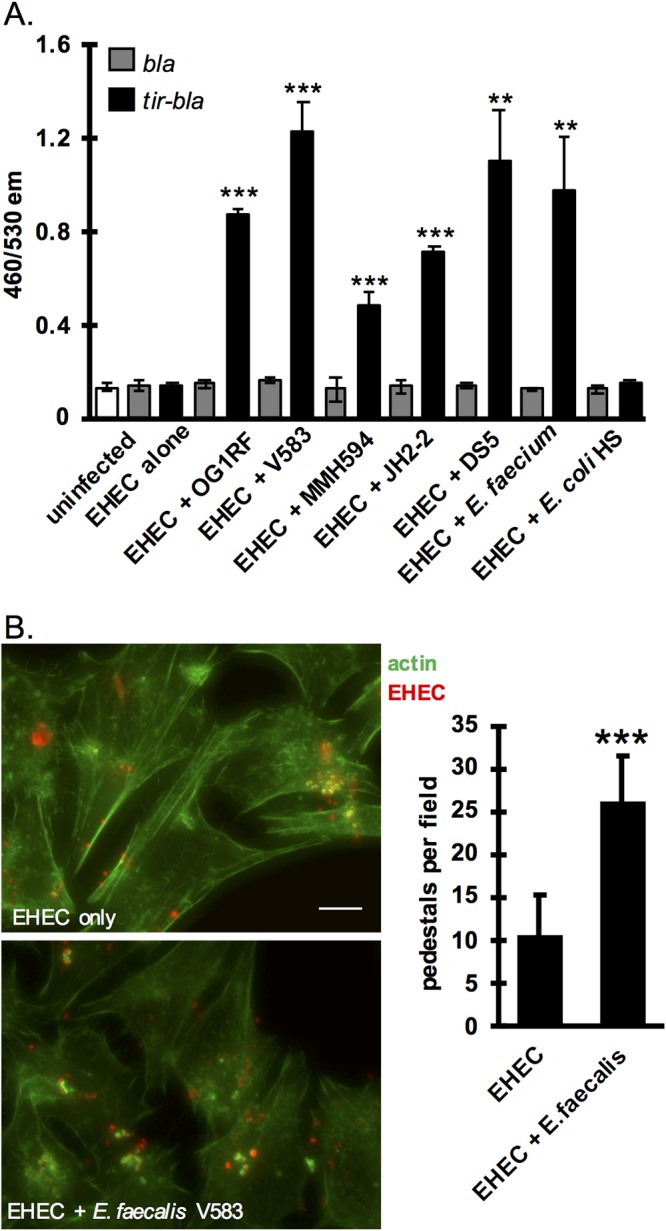
E. faecalis enhances EHEC T3SS activity. (A) A TEM-1 β-lactamase translocation assay was used to measure translocation of Tir. Reporter EHEC strains express either TEM-1 β-lactamase (*bla*) as a control or a *tir-bla* fusion that will be translocated into host cells via the T3SS. EHEC reporter strains were pregrown under T3SS-inducing conditions alone or in the presence of the indicated commensal strains and then used to infect HeLa cells. HeLa cells were then loaded with a fluorescent β-lactam compound whose emission spectrum is altered by β-lactamase cleavage. The 460/530 emission (em) ratio reflects the level of β-lactamase activity and therefore the level of Tir translocation into cells. The average and standard deviation of three replicates from a single experiment are plotted, and the experiment was repeated three times. (B) HeLa cells were infected with EHEC with or without E. faecalis strain V583 for 6 h; then cells were washed, fixed, and stained with FITC-phalloidin. Pedestals were visualized as green puncta of actin beneath attached red bacteria. The numbers of pedestals per field were counted for seven randomly selected fields, and the average and standard deviations are plotted. Scale bar, 10 μm. Two-tailed *t* tests were performed to determine significant differences compared to results for EHEC alone. **, *P* < 0.01; ***, *P* < 0.001.

The mechanism underlying enhanced LEE transcription in the presence of B. thetaiotaomicron has been described previously (6); however, the mechanism underlying the effect of E. faecalis on LEE transcription is unknown, and we carried out the experiments described below to characterize the interaction between EHEC and E. faecalis further. This interaction is of particular interest as, in mice, E. faecalis can directly colonize the intestinal epithelium (16), the site where the EHEC T3SS is deployed, while both commensal E. coli HS and B. thetaiotaomicron are thought to dwell primarily in the intestinal lumen or outer mucus layer (18–20). We first investigated whether increased LEE transcription in the presence of E. faecalis translated into increased activity of the EHEC T3SS. We measured T3SS activity via two readouts: translocation of the effector protein Tir and pedestal formation. To measure the levels of effector protein injected into host cells by the T3SS, we performed a TEM-1 β-lactamase (βla)-based translocation assay (21). In this assay HeLa cells are infected with an EHEC reporter strain expressing a βla-Tir fusion protein, and then HeLa cells are loaded with a fluorescent βla substrate whose emission spectrum is altered upon cleavage. The amount of Tir translocation can then be measured as the ratio of cleaved (emission at 460 nm) to uncleaved (emission at 530 nm) substrate. An EHEC strain expressing an unfused βla, which should not be translocated, was included as a negative control. In the presence of E. faecalis strain V583, Tir translocation was significantly increased compared to that for EHEC alone (Fig. 2A). To investigate whether the effect of E. faecalis was universal or strain specific, four more strains of E. faecalis were tested, OG1RF, MMH594, JH2-2, and DS-5, as well as one strain of Enterococcus faecium. E. coli HS was included as a control as it had no effect on LEE transcription. Whether the strain was originally isolated from a healthy individual (OG1RF) or a hospital patient (all other strains), all strains of E. faecalis plus the single strain of E. faecium tested significantly enhanced translocation of Tir compared to that for EHEC alone (Fig. 2A). This suggests that the mechanism by which *Enterococcus* enhances LEE expression is not strain specific but may be a general characteristic of enterococci. Interestingly, there were some significant differences between strains in the magnitudes of their enhancing effect on Tir translocation. For example, E. faecalis MMH594, a highly virulent disseminated hospital strain, showed the smallest enhancement of Tir translocation of all strains. This is a notable observation that is not explored further in this work but may be of interest for future studies. No significant differences in growth were observed between any of the E. faecalis strains tested (data not shown). In agreement with the qPCR data, the presence of commensal E. coli HS did not increase translocation of Tir.

Injection of EHEC T3SS effector proteins into host cells causes host actin rearrangement localized around the attached bacterium, resulting in a pedestal-like structure that is a hallmark of attaching and effacing pathogens like EHEC (11). To enumerate pedestal formation, we infected HeLa cells with EHEC expressing mCherry in the presence or absence of E. faecalis V583 and performed a fluorescent actin staining (FAS) assay. Cells were stained with fluorescein isothiocyanate (FITC)-phalloidin, and pedestals were visualized as bright green puncta of polymerized actin beneath attached red bacteria. The average number of pedestals per field of cells was more than doubled in the presence of E. faecalis compared with the level for EHEC alone (Fig. 2B). HeLa cells are routinely used in the field to quantify pedestal formation by EHEC because this cell type supports robust pedestals that are relatively discrete and readily quantified (22–24). We performed these experiments on a more relevant cell type as well, HT-29 intestinal epithelial cells, and similar patterns were observed, with apparently more actin polymerization observed in the presence of E. faecalis (Fig. S2). However, because of the morphology of the pedestals formed on this cell type, they could not be quantified with confidence.

10.1128/mBio.02547-19.2 FIG S2 E. faecalis increases EHEC pedestal formation on HT-29 intestinal epithelial cells. Fluorescent actin staining (FAS) assay was performed on HT-29 intestinal epithelial cells. Confluent monolayers of HT-29 cells were infected with either EHEC expressing mCherry, E. faecalis strain OG1RF, or both. Initial EHEC/E. faecalis ratios of 1:1 and 1:10 were tested. After 6 hours infected cells were fixed, permeabilized, and stained with FITC-phalloidin and Hoechst. E. faecalis cells are stained with Hoechst dye, but because of the much brighter staining of host nuclei, the bacterial cells are difficult to visualize. Similar patterns are observed for infections performed on HeLa cells (more actin polymerization observed in the presence of E. faecalis); however, the morphology of the pedestals formed on the HT-29 cell type makes them difficult to quantify. No pedestal-like structures were observed when HT-29 cells were infected with E. faecalis alone. For each condition one representative field of view at 63× magnification is shown along with an area of interest (indicated by white box) scaled to 200% to allow better visualization of pedestal morphology. Download FIG S2, TIF file, 2.7 MB. Copyright © 2019 Cameron et al. 2019 Cameron et al. This content is distributed under the terms of the Creative Commons Attribution 4.0 International license.

The results of the FAS and Tir translocation assays support the idea that the increased LEE transcription caused by E. faecalis translates to increased activity of this important virulence factor. For all future experiments, except where otherwise stated, the OG1RF strain of E. faecalis was used because it displays a robust phenotype similar to that observed with the V583 strain but lacks many of the accessory plasmids and virulence factors carried by V583 and is more readily manipulated genetically (25).

### EspB protein is degraded in coculture with E. faecalis.

**FIG 3:**
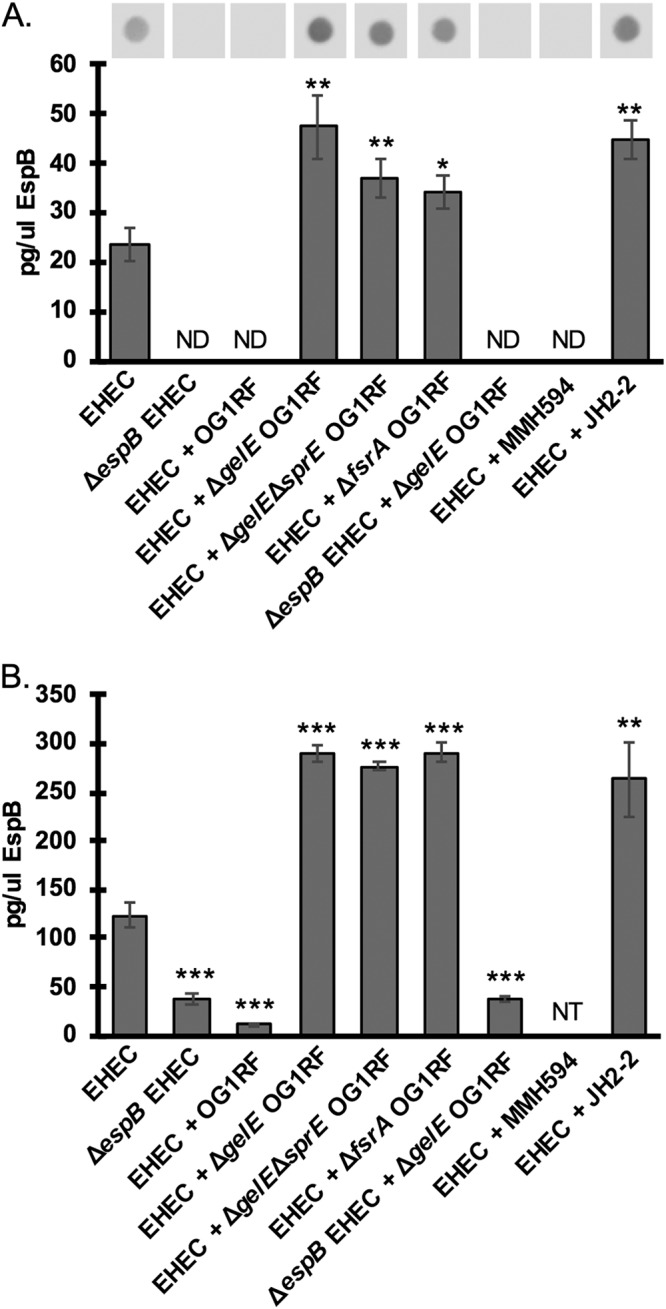
The E. faecalis protease GelE degrades EHEC EspB. (A) EspB protein levels from EHEC cultures with or without E. faecalis measured by dot blot assay. Intensity of the spots was measured using ImageJ and converted to absolute EspB concentration using a standard curve of known recombinant EspB concentrations included on the same blot. The average and standard deviation of three replicates from one experiment are plotted here, and the experiment was repeated three times. ND, not detected (insufficient signal). (B) EspB protein levels from EHEC cultures with or without E. faecalis measured by ELISA. EspB concentrations in samples were calibrated to a standard curve of known recombinant EspB concentrations. The average and standard deviation of three technical replicates are plotted here, and the experiment was repeated twice. NT, not tested. Two-tailed *t* tests were performed to determine significant differences compared to results for EHEC alone. *, *P* < 0.05; **, *P* < 0.01; ***, *P* < 0.001.

To monitor expression of the EHEC T3SS at the protein level, we developed a dot blot assay to measure the levels of one of the major structural proteins of the T3SS, EspB (Fig. 1A). EspB, along with EspD, is involved in pore formation in the host cell membrane (26), and we hypothesized that it would be a good protein target to monitor changes in the T3SS because it is cotranscribed with *espA*, the gene most highly upregulated by the presence of E. faecalis, as measured by our qPCR assay (Fig. 1B). Surprisingly, while EspB from EHEC monocultures was readily detectable by dot blot assay, EspB was undetectable in cocultures of EHEC and E. faecalis, which showed a phenotype similar to that of an EHEC Δ*espB* mutant strain (Fig. 3A). An enzyme-linked immunosorbent assay (ELISA) measuring EspB protein was also performed to confirm these results, and, again, EspB was completely lost from cocultures of EHEC and E. faecalis, which had protein levels indistinguishable from the level of the EHEC Δ*espB* mutant strain (Fig. 3B).

### EspB is cleaved by the E. faecalis protease GelE.

Because of the disconnect between the increased transcription of the LEE and the undetectable EspB protein levels in EHEC and E. faecalis cocultures, we hypothesized that EspB may be actively degraded in the presence of E. faecalis. Indeed, previous studies have shown that EspB is particularly susceptible to proteolytic cleavage and can be targeted by EHEC and commensal proteases (17, 27). E. faecalis produces two major secreted proteases: gelatinase (GelE) and serine protease (SprE). GelE and SprE are important for E. faecalis biofilm formation (28, 29), and GelE plays a role in autolysis (30) and chain length determination (31) and increases virulence in an E. faecalis endocarditis model of disease (32). When EHEC was cocultured with a Δ*gelE* mutant strain of E. faecalis, EspB protein levels were again detectable (Fig. 3A and B), suggesting that GelE is indeed degrading secreted EspB. In fact, EspB levels in coculture with the Δ*gelE* mutant strain were increased compared to the level for EHEC alone, likely due to the increased transcription of the LEE in the presence of E. faecalis (Fig. 1B), which then gives rise to increased levels of secreted EspB when GelE degradation of EspB is abrogated. Coculture with a Δ*gelE* Δ*sprE* double mutant resulted in EspB levels similar to those of the Δ*gelE* single mutant, suggesting that EspB proteolytic degradation occurs mainly via GelE (or possibly another protease activated by GelE).

Expression of *gelE* and *sprE* is positively regulated by the *fsr* quorum sensing system (33, 34). A mutant with a deletion of the response regulator of this system (Δ*fsrA*) behaved similarly to the Δ*gelE* mutant, with increased EspB protein levels compared to the level in cultures of EHEC alone. This supports the hypothesis that GelE is responsible for the observed degradation of EspB. Additionally, because levels of EspB protein are higher in the presence of the *fsrA* mutant (Fig. 3) than in cultures of EHEC alone, we also hypothesize that the original enhancing effect of E. faecalis shown in Fig. 1 and 2 is not dependent on *fsr* quorum sensing by E. faecalis. To control for antibody specificity, we included a coculture of Δ*espB* EHEC and Δ*gelE* E. faecalis. No significant signal was detected under this condition with either the dot blot assay or ELISA, confirming that there is no significant cross-reactivity between the EspB antibody and other proteins present in the cocultures (Fig. 3A and B).

We measured EspB levels from cocultures of EHEC with two independent isolates of E. faecalis that naturally differ in their expression of the *fsr* quorum sensing system. MMH594, like the OG1RF strain, has an intact *fsr* system and is phenotypically GelE^+^, whereas strain JH2-2 does not have a functional *fsr* system and is therefore phenotypically GelE^−^. Similar to results with wild-type (WT) OG1RF, coculture with MMH594 (GelE^+^) resulted in complete loss of EspB from culture supernatants (Fig. 3A), whereas coculture with JH2-2 (GelE^−^) resulted in EspB levels similar to (Fig. 3A) or elevated (Fig. 3B) compared to those for EHEC alone. The cumulative results show that *fsr*-dependent production of secreted GelE degrades EspB and that production of the E. faecalis factor mediating increased LEE expression occurs independently from the known enterococcal quorum sensing system.

As EHEC and E. faecalis are both facultative anaerobes, we performed cocultures under aerobic and anaerobic conditions to test whether the oxygen environment would impact the effect of E. faecalis on EHEC LEE expression. Neither the ability of E. faecalis to increase EspB expression nor the degradation of EspB by GelE appeared to be affected by the level of oxygen in the growth environment (Fig. S3).

10.1128/mBio.02547-19.3 FIG S3 EspB degradation by GelE occurs in anaerobic conditions. EHEC was grown alone or with the indicated E. faecalis strains in anaerobic conditions for 6 hours, and EspB protein levels were quantified by ELISA. The average and standard deviation of three technical replicates are plotted here, and the experiment was repeated twice. A two-tailed *t* test was performed to determine significant differences compared to results with EHEC alone. *, *P* < 0.05; **, *P* < 0.01; ***, *P* < 0.001. Download FIG S3, TIF file, 1.1 MB. Copyright © 2019 Cameron et al. 2019 Cameron et al. This content is distributed under the terms of the Creative Commons Attribution 4.0 International license.

We also tested whether the ratio of EHEC to E. faecalis impacted the ability of E. faecalis to increase LEE expression by EHEC. Using a dot blot assay to measure EspB expression by EHEC, we determined that both a 1:1 and 1:10 ratio of EHEC to E. faecalis resulted in increased EspB expression by EHEC compared to that when EHEC was cultured alone. A 1:10 ratio of EHEC to E. faecalis increased EspB concentration approximately 5-fold compared to that for EHEC alone where the 1:1 ratio resulted in an approximately 3-fold increase in EspB concentration (Fig. S4). This suggests that the ability of E. faecalis to increase LEE expression in EHEC does not require a strict ratio of the two bacteria and that generally larger numbers of E. faecalis generate a stronger effect. For the remaining experiments, except where otherwise stated, an initial ratio of 1:1 of EHEC to E. faecalis was used.

10.1128/mBio.02547-19.4 FIG S4 Increasing the ratio of E. faecalis to EHEC increases the effect on LEE expression. EHEC was grown under T3SS-inducing conditions (DMEM with 1g/liter glucose) with E. faecalis at either a 1:1 or 1:10 ratio, and the resulting level of EspB protein was quantified by dot blot assay. To facilitate the use of the EspB dot blot assay, the Δ*gelE* strain of OG1RF E. faecalis was used to remove the confounding effect of GelE cleavage of EspB. The average and standard deviation of three biological replicates are shown. A student’s *t* test was performed to assess significant differences between results for the indicated conditions. ***, *P* < 0.001. Download FIG S4, TIF file, 0.9 MB. Copyright © 2019 Cameron et al. 2019 Cameron et al. This content is distributed under the terms of the Creative Commons Attribution 4.0 International license.

### GelE degradation of EspB enhances Tir translocation by EHEC.

**FIG 4:**
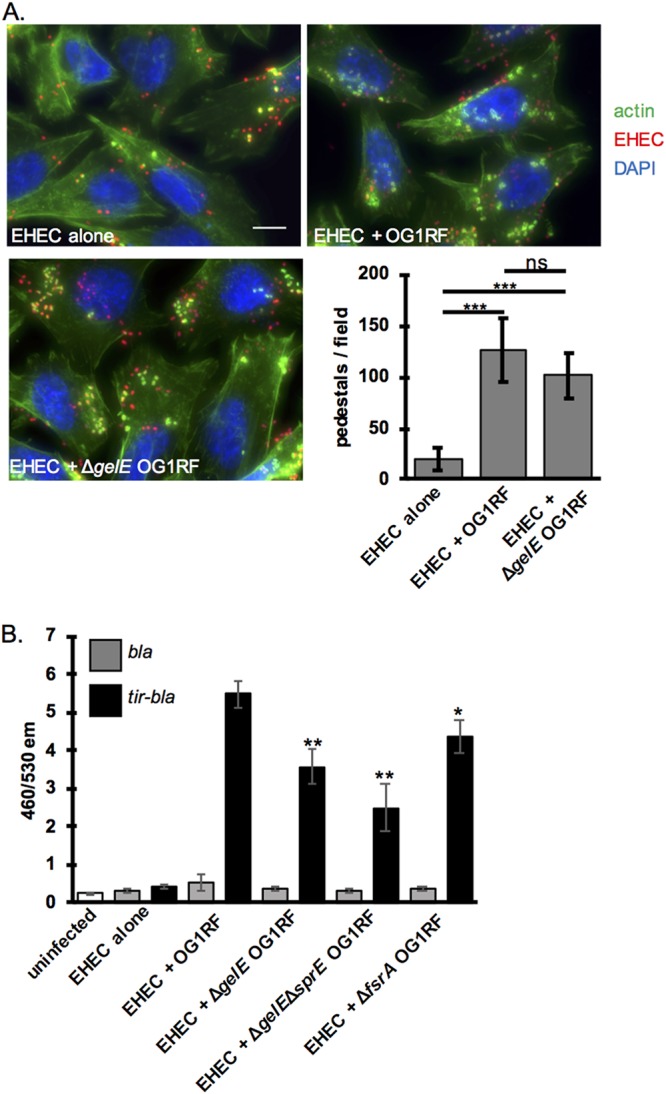
GelE degradation of EspB affects EHEC T3SS function. (A) FAS assay to visualize EHEC pedestal formation in the presence of WT versus Δ*gelE* E. faecalis. HeLa cells infected by mCherry expressing EHEC (red) were stained with FITC-phalloidin to visualize actin (green) and DAPI to stain DNA (blue). Representative images from a single experiment are shown. The numbers of pedestals per field were counted for six randomly selected fields, and the average and standard deviations across fields for a single experiment are shown. The experiment was repeated three times, and the same patterns were observed. Scale bar, 10 μm. Two-tailed *t* tests were performed to determine significant differences between results for the conditions indicated. (B) Tir translocation was measured via the assay described in the legend of Fig. 1D in the presence of WT versus Δ*gelE* E. faecalis. The average and standard deviations for three replicates from a single experiment are plotted, and the experiment was repeated three times. Two-tailed *t* tests were performed to determine significant differences compared to results for EHEC alone. *, *P* < 0.05; **, *P* < 0.01; ***, *P* < 0.001; ns, not significant.

EspB is required for activity of the EHEC T3SS (35). To investigate the effect of GelE on EHEC pedestal formation, we performed a FAS assay to visualize pedestal formation. Despite its capacity to degrade EspB, the presence of wild-type E. faecalis increased the number of pedestals formed on EHEC-infected HeLa cells, as seen previously (Fig. 2B). While wild-type OG1RF appeared to enhance pedestal formation more than Δ*gelE* E. faecalis, the difference was not statistically significant (Fig. 4A). Similarly, the Δ*gelE* Δ*sprE* and the Δ*fsrA* mutant strains of OG1RF appeared to increase EHEC pedestal formation to the same degree as the WT OG1RF strain (Fig. S5).

10.1128/mBio.02547-19.5 FIG S5 EHEC pedestal formation in the presence of various E. faecalis mutants. FAS assay to visualize EHEC pedestal formation in the presence of WT or mutant E. faecalis. HeLa cells infected by mCherry expressing EHEC (red) were stained with FITC-phalloidin to visualize actin (green) and with DAPI to stain DNA (blue). Representative images from a single experiment are shown. Pedestals per field were counted for six individual fields, and the average and standard deviations across fields for a single experiment are shown. A two-tailed *t* test was performed to assess significant differences compared to results with EHEC alone. The experiment was repeated three times, and the same patterns were observed. Scale bar, 10 μm. *, *P* < 0.05; **, *P* < 0.01; ***, *P* < 0.001. Download FIG S5, TIF file, 2.4 MB. Copyright © 2019 Cameron et al. 2019 Cameron et al. This content is distributed under the terms of the Creative Commons Attribution 4.0 International license.

We also investigated the effect of GelE on Tir translocation, a further upstream activity of the EHEC T3SS. Although Tir translocation is generally enhanced in the presence of E. faecalis (Fig. 2A and 4B), this was reduced when the strain used was GelE^−^(Δ*gelE*, Δ*gelE* Δ*sprE*, and Δ*fsrA* mutant strains of E. faecalis) compared to the level with a GelE^+^ wild-type strain (Fig. 4B). This suggests that GelE cleavage of EspB may actually enhance activity of the EHEC T3SS. The E. faecalis-EHEC interaction appears to be multifaceted as this experiment also demonstrates that E. faecalis enhances EHEC T3SS activity via a mechanism independent of GelE because GelE^−^ strains of E. faecalis still increased Tir translocation compared to the level for EHEC alone. We hypothesize that this GelE-independent mechanism is driving the increased LEE transcription demonstrated in Fig. 1B.

### The effect of E. faecalis on EHEC LEE expression is dependent on cell contact.

**FIG 5:**
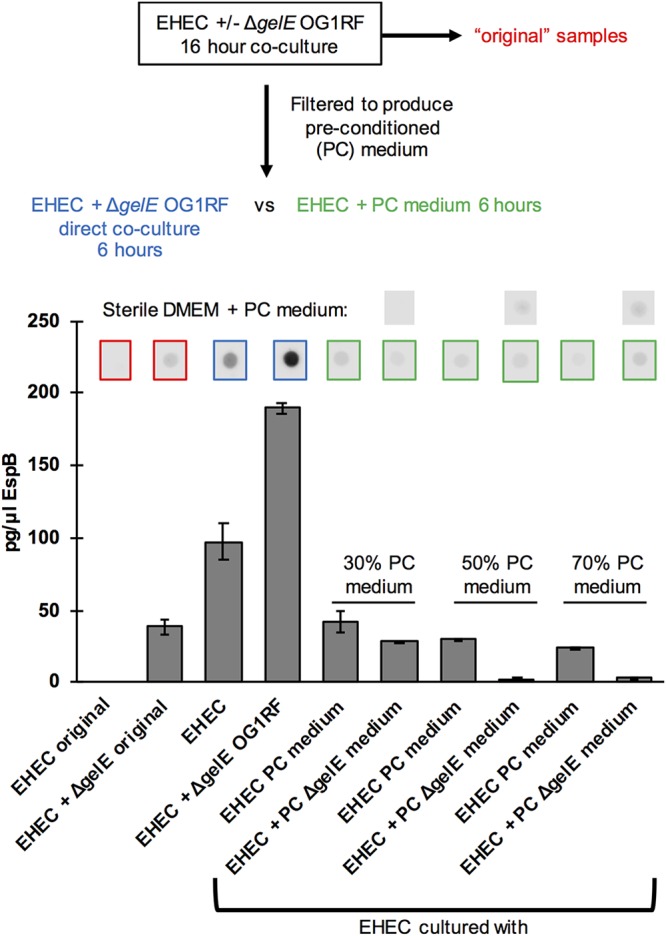
E. faecalis-mediated increased LEE expression requires cell contact. A schematic of the experiment performed is shown. EHEC was cultured alone or with Δ*gelE* E. faecalis for 16 h. Samples from these initial cultures were included on the final dot blot, designated original cultures, and also filtered to generate preconditioned (PC) medium for subsequent EHEC treatment. EHEC was then grown alone, in direct coculture with E. faecalis, or in the presence of preconditioned medium from EHEC alone (negative control) or EHEC and E. faecalis coculture for 6 h. Three different concentrations of preconditioned medium were tested (30%, 50%, and 70%). Sterile DMEM was mixed with preconditioned medium in the correct ratios to allow for subtraction of EspB signal originating from the preconditioned medium itself. A dot blot assay for EspB was performed, spot intensity from three replicate spots was quantified, and the average and standard deviation from one experiment are plotted. The experiment was repeated three times to ensure reproducibility.

One potential mechanism by which E. faecalis may impact LEE expression is production of a diffusible chemical signal which is then sensed by EHEC, similar to how B. thetaiotaomicron succinate production impacts EHEC LEE expression (6). To test whether the effect of E. faecalis on EHEC LEE expression required cell contact, which would not be required by a freely diffusible signal, EHEC cocultured with E. faecalis was compared to EHEC exposed to preconditioned medium from a coculture of EHEC and E. faecalis (Fig. 5). EHEC and E. faecalis coculture was used to generate the preconditioned medium because it is possible that the putative E. faecalis signal is produced only in the presence of EHEC. A Δ*gelE* mutant strain of E. faecalis was used in these experiments to remove the confounding effect of EspB degradation by GelE and to allow us to use the EspB dot blot assay to monitor LEE expression. To generate preconditioned medium, EHEC was grown with or without Δ*gelE* E. faecalis under T3SS-inducing conditions; then cells were pelleted, and the supernatant was filtered. Samples from these cultures (designated original) were included on the dot blot to ensure that LEE expression was enhanced in the presence of E. faecalis in these cultures and therefore would contain the putative soluble signal. A second round of cocultures was then performed comparing EHEC grown alone, EHEC directly cocultured with Δ*gelE* E. faecalis, or EHEC exposed to preconditioned medium from a culture of EHEC alone or of EHEC plus Δ*gelE* E. faecalis. To control for and subtract EspB signal originating from the preconditioned medium, sterile medium was combined with preconditioned medium in appropriate ratios and included on the dot blot such that only EspB originating from actively growing EHEC was quantified. When EHEC was directly cocultured with Δ*gelE* E. faecalis, a clear increase in EspB protein levels compared to the level for EHEC alone was observed, as expected. However, there was no increase in EspB protein levels in EHEC cultures exposed to preconditioned medium from EHEC and Δ*gelE* E. faecalis cocultures compared to the level for EHEC exposed to preconditioned medium from cultures of EHEC alone. Three different ratios of fresh medium to preconditioned medium were tested (30%, 50%, and 70% preconditioned medium), and none resulted in an increase in EspB levels. In fact, when the EspB signal originating from the preconditioned medium itself is subtracted, EspB levels are diminished in cultures exposed to higher concentrations of preconditioned medium from cultures of EHEC plus Δ*gelE* E. faecalis compared to levels for cultures exposed to medium from EHEC alone (Fig. 5). This experiment suggests that the E. faecalis signal leading to increased LEE expression is not present in cell-free medium of cultures that were themselves induced and therefore supports a model whereby cell contact between EHEC and E. faecalis is necessary for the inducing effect of E. faecalis.

## DISCUSSION

Here, we demonstrate that the commensal bacterium Enterococcus faecalis impacts the activity of the EHEC T3SS via two distinct mechanisms (see Fig. S6 in the supplemental material): (i) a transcriptional effect and (ii) a posttranslational effect. E. faecalis enhances transcription of the LEE pathogenicity island via a mechanism that is dependent on cell contact between E. faecalis and EHEC. This is in marked contrast to the previously reported mechanism by which B. thetaiotaomicron increases LEE expression, via secretion of succinate, which is then sensed by EHEC (6). The contrast is particularly interesting considering where these commensals are spatially distributed in the gastrointestinal tract. *Bacteroides* resides primarily in the lumen of the large intestine or outer mucus layer (18, 19) where EHEC LEE expression is undesirable, whereas E. faecalis readily colonizes the intestinal epithelium of germ-free mice (16). Upregulation of the LEE upon cell contact with E. faecalis would be a mechanism by which EHEC could tune expression of this energetically expensive system to be expressed only when it is in very close proximity to the epithelium. Notably, epithelial colonization by *Enterococcus* in conventional animals has not yet been demonstrated, nor has it been shown that *Enterococcus* and EHEC colocalize *in vivo*. This will be the focus of future work as will identification of E. faecalis genes that are involved in modulating LEE expression and determining the precise mechanism of this interaction.

10.1128/mBio.02547-19.6 FIG S6 Model for E. faecalis-EHEC interactions in the intestine. Both E. faecalis (purple cocci) and EHEC (pink rods) can colonize the epithelial cell (beige) surface, with the potential to form biofilms. (Interaction 1) When the two species are in close proximity, a signal from E. faecalis activates LEE locus transcription, increasing production of the T3SS. The T3SS proteins form a needle-like structure (blue) with EspB/D at the tip (red). (Interaction 2) GelE protease from adjacent E. faecalis cells processes EspB (and possibly other T3SS components), increasing the ability of the secretion machine to translocate effectors into the host cell. Effector proteins cause actin polymerization, ultimately leading to formation of pedestals and enhance EHEC attachment. Download FIG S6, TIF file, 0.9 MB. Copyright © 2019 Cameron et al. 2019 Cameron et al. This content is distributed under the terms of the Creative Commons Attribution 4.0 International license.

We also demonstrate that the E. faecalis protease GelE cleaves a critical component of the EHEC T3SS, EspB. Our experiments support the idea that GelE cleavage of EspB actually enhances activity of the EHEC T3SS, adding to a growing body of work suggesting that proteolytic cleavage regulates activity of the T3SSs of enteropathogenic E. coli (EPEC) and EHEC. Studies show that endogenously expressed proteases EspP and EspC, from EHEC and EPEC, respectively, negatively regulate activity of the T3SS (17, 27), while exogenous proteases from B. thetaiotaomicron (17), and now E. faecalis, appear to enhance activity of the system. This presents an intriguing model whereby the T3SS is regulated posttranslationally by proteolytic cleavage, and the balance of endogenous versus exogenous proteases that are present alters the activity of the system. The mechanism by which protease cleavage alters activity of the T3SS remains unclear, but we can speculate on a few potential mechanisms. The simplest model is that proteolytic processing promotes pore formation by EspB, as has been observed with other pore-forming proteins, like the protective antigen component of anthrax toxin (36). It is also possible that proteolytic degradation of one or more structural proteins of the T3SS may alter their stoichiometry to optimize activity of the system. Finally, to our knowledge, a mechanism for disassembly of the T3SS after effector injection has not been described. However, this step is seemingly necessary to facilitate binding between Tir on the target host cell surface and intimin on the bacterial outer membrane. It is tempting to speculate that degradation of T3SS structural proteins by proteases like GelE may play a role in disassembling the T3SS after effector injection to promote Tir-intimin binding and tight adherence.

In order for the secreted proteases of a commensal to have a significant effect on the EHEC T3SS, the two bacteria would need to be in relatively close proximity. The ability of E. faecalis to colonize in close association with the host epithelium (16), where the EHEC T3SS is deployed, makes it a particularly attractive candidate commensal for this model. The T3SS virulence factor is used by many human pathogens, such as *Salmonella*, *Shigella*, *Yersinia*, and others, and the structural homology across these systems suggests that this model of cross-species proteolytic processing may apply to systems in other species as well.

The phenomenon of a commensal increasing virulence of a pathogen raises the question of whether the commensal species stands to benefit from this interaction. An interesting future line of research will be to investigate whether enterococcal populations are increased during an EHEC infection or intestinal infection in general. It is tempting to speculate that E. faecalis, an opportunistic pathogen itself, may bloom in the diseased environment created during EHEC infection, thus creating a mutually beneficial relationship.

## MATERIALS AND METHODS

### Strains, plasmids, and culture conditions.

Strains and plasmids used in this work are listed in Table S1 in the supplemental material. EHEC was routinely grown in LB medium and under conditions where expression of the T3SS was desirable. Dulbecco’s modified Eagle’s medium with 1 g/liter glucose (DMEM-low glucose) was used. E. faecalis strains were routinely cultured in brain heart infusion (BHI) medium. Anaerobic growth was performed using a GasPak EZ anaerobe container system (Becton, Dickinson). HeLa cells were routinely cultured in complete DMEM (cDMEM), defined as DMEM with 4.5 g/liter glucose, 10% fetal bovine serum (FBS), and penicillin/streptomycin/glutamine.

10.1128/mBio.02289-19.7 TABLE S1 Strains and plasmids. Download Table S1, DOCX file, 0.01 MB. Copyright © 2019 Cameron et al. 2019 Cameron et al. This content is distributed under the terms of the Creative Commons Attribution 4.0 International license.

### qRT-PCR.

DMEM with 1 g/liter glucose was inoculated at 1:100 with overnight cultures of EHEC, B. thetaiotaomicron, E. faecalis, or E. coli HS. Before inoculation, commensals were concentrated 10× such that they were added at 10-fold excess over EHEC to mimic ratios in the gut. Cultures were grown for 4 h to late log phase when EHEC CFU counts were comparable between cultures. RNA was extracted using a Ribopure bacteria isolation kit (Ambion) according to the manufacturer’s protocols. Primers used for quantitative reverse transcription-PCR (qRT-PCR) (Table S2) were validated for amplification efficiency and template specificity. qRT-PCR was performed as described in Hughes et al. (37) using a one-step reaction on an ABI 7500 sequence detection system, and data were collected using ABI Sequence Detection, version 1.2, software (Applied Biosystems). Values were normalized to the level of endogenous *rpoA* expression for cultures using WT EHEC and the engineered chloramphenicol (Cm) resistance gene for cultures using the *rpsMp_*Cm*::LacZ* EHEC strain (see below). Normalized gene expression values were analyzed using the comparative critical threshold method. Values are presented as fold change over WT levels, and the average and standard deviation of four independent replicates are represented.

10.1128/mBio.02289-19.8 TABLE S2 Primers. Download Table S2, DOCX file, 0.01 MB. Copyright © 2019 Cameron et al. 2019 Cameron et al. This content is distributed under the terms of the Creative Commons Attribution 4.0 International license.

### Engineering a unique housekeeping gene into EHEC.

In order to quantify changes in virulence gene expression in cocultures of EHEC and E. coli HS, it was necessary to engineer a unique housekeeping gene into EHEC because the sequence divergence of common housekeeping genes between the two strains was insufficient to allow for specific amplification from one strain for normalization. To this end, the EHEC promoter driving *rpoA* expression (146 bp upstream of the *rpsM* start codon) was cloned upstream of a chloramphenicol resistance gene (from pKD3) and inserted into the genome between the *lacI* and *lacZ* genes using lambda red recombination to create the *rpsMp_*Cm*::LacZ* EHEC strain. qPCR primers directed toward the chloramphenicol resistance gene were designed, and expression levels of this engineered housekeeping gene were used to normalize values for the genes of interest. This approach was validated by comparing the relative fold change of *espA* expression at 6 and 8 h of growth to the level at 4 h of growth under T3SS-inducing conditions. *espA* transcript levels were normalized to Cm transcript levels in the *rpsMp*_Cm::*LacZ* EHEC strain and *rpoA* transcript levels in the WT EHEC strain. *espA* fold changes measured at 6 and 8 hours were not significantly different between the two strains using the respective normalization methods.

### Fluorescent actin staining (FAS) assay.

HeLa cells were grown on coverslips in 12-well culture plates in cDMEM at 37°C in 5% CO_2_ overnight to approximately 80% confluence. The wells were washed with phosphate-buffered saline (PBS) and replaced with low-glucose DMEM. EHEC containing the mCherry expression plasmid pDP151 was grown as standing cultures overnight in LB medium with ampicillin, and E. faecalis cultures were grown as standing cultures overnight in BHI medium. Bacterial cultures were then diluted 1:100 into wells containing HeLa cells for 6 h at 37°C in 5% CO_2_, with medium being removed and replaced at 3 h postinfection. After a 6-h infection, the coverslips were washed, fixed with formaldehyde, permeabilized, and treated with fluorescein isothiocyanate (FITC)-labeled phalloidin to visualize actin accumulation. Coverslips were mounted on slides with ProLong Gold Antifade mountant or Prolong Gold with 4′,6′-diamidino-2-phenylindole (DAPI; Molecular Probes) to visualize host nuclei. Samples were visualized with a Zeiss LSM800 confocal microscope using a 63× objective and 405-, 488-, and 640-nm excitation lasers. The number of pedestals per field was enumerated for each field of cells, and the average and standard deviation across seven fields are displayed for one experiment. Experiments were repeated three times, and differences between experimental groups were consistent. However, because of differences in overall pedestal levels, results could not be averaged across experiments.

### Tir translocation assay.

HeLa cells were seeded in at 1 × 10^4^ cells per well into a black-walled, clear-bottom 96-well plate 48 h prior to infection in cDMEM. Overnight bacterial cultures were subcultured 1:20 into 1.5 ml of DMEM-low glucose and 5% FBS in a 12-well plate. Cultures were grown for 3 h at 37°C in 5% CO_2_. Medium was removed from HeLa cells, cells were washed once with sterile DMEM-low glucose medium, and then 100 μl of DMEM-low glucose plus 5% FBS was added to each well. HeLa cells were infected with 15 μl of the pregrown bacterial cultures and incubated at 37°C in 5% CO_2_ for 30 min. Blank (no HeLa cells) and uninfected wells were included as controls, and each experimental condition was run in triplicate. Reporter constructs were then induced by the addition of 0.2% arabinose (final concentration) and incubation for 1 h. Medium was then removed from cells, cells were washed once with Hanks balanced salt solution (HBSS), and cells were loaded with freshly prepared coumarin cephalosporin fluorescein acetoxymethyl ester (CCF2-AM) substrate according to manufacturer’s instructions using a GeneBLAzer *in vivo* detection kit (Invitrogen). Results were read on a Synergy H1 Hybrid Multi-Mode plate reader (Biotek). Readings were collected from the bottom by excitation at 405 nm and reading emission at 460 nm and 530 nm and using the autogain function. For each well the ratio of 460/530-nm emission was calculated, and then average and standard deviations for the three replicate wells were calculated. Experiments were repeated three times, and differences between experimental groups were consistent. However, because of differences in overall translocation levels, results could not be averaged across experiments.

### Dot blot assay.

Cocultures were performed in triplicate in 150 μl of DMEM with 1 g/liter glucose in a polypropylene 96-well plate (Nunc). WT or mutant EHEC bacteria were inoculated 1:100 from overnight cultures grown in LB medium, and WT or mutant E. faecalis bacteria were inoculated 1:100 from overnight cultures grown in BHI medium. Plates were grown for 6 h in a 37°C, 5% CO_2_, cell culture incubator in a water bath. Plates were then heated to 95°C for 10 min to kill bacteria. A standard curve of purified recombinant EspB protein was included so that absolute EspB concentrations could be determined. Heat-inactivated samples (100 μl) and standards were applied to a 0.2-μm-pore-size nitrocellulose membrane using a Bio-Dot dot blot apparatus (BioRad) according to manufacturer’s instructions. EspB protein was detected using a rabbit anti-EspB polyclonal antibody (1:10,000) and a goat anti-rabbit horseradish peroxidase (HRP)-conjugated secondary antibody (1:20,000). Dot blots were developed using SuperSignal West Pico Plus chemiluminescent substrate (Thermo) and imaged on a LiCor Odyssey FC imaging system. Intensity of the spots was quantified using ImageJ.

### EspB ELISA.

EspB ELISA was performed as described in Pifer et al. (38). Briefly, 150-μl cultures of EHEC with or without E. faecalis were grown in 96-well plates aerobically or anaerobically for 6 h. Cultures were inactivated by adding sodium azide and a protease inhibitor cocktail (P8849; Sigma). A standard curve of recombinant EspB protein was treated as the samples were and included so that absolute values of samples could be determined. Samples (100 μl) and standards were applied to MaxiSorp ELISA plates (Nunc), and EspB was detected using a rabbit anti-EspB polyclonal antibody (1:1,000) and a goat anti-rabbit HRP-conjugated secondary antibody (1:1,000). Plates were developed using a 3,3′,5,5′-tetramethylbenzidine (TMB) liquid substrate system (T0440; Sigma), and the reaction was stopped with HCl. Absorbance at 450 nm was read in a Synergy H1 plate reader (BioTek). The average and standard deviation across three replicates for a single experiment are shown. The experiment was repeated three times with similar results.

### Treatment of EHEC with preconditioned culture medium.

DMEM with 1 g/liter glucose was inoculated 1:100 with overnight cultures of EHEC with or without Δ*gelE* OG1RF allowed to grow for 16 h in 5% CO_2_ at 37°C. After 16 h of growth, samples were collected and boiled for 10 min (to produce original samples); the remaining culture was centrifuged, and the supernatant was filtered through a 0.2-μm-pore-size polyvinylidene difluoride (PVDF) filter to produce the preconditioned medium. Fresh overnight cultures of EHEC were diluted 1:100 into fresh DMEM with 1 g/liter glucose or a mixture of fresh DMEM and preconditioned medium. A new direct coculture of EHEC and Δ*gelE* OG1RF in DMEM with 1 g/liter glucose was also performed. The growth and dot blot assays were then performed as described above.

## References

[1] Baumler AJ, Sperandio V 2016 Interactions between the microbiota and pathogenic bacteria in the gut. Nature 535:85–93. doi:10.1038/nature18849. 27383983 PMC5114849

[2] Lupp C, Robertson ML, Wickham ME, Sekirov I, Champion OL, Gaynor EC, Finlay BB 2007 Host-mediated inflammation disrupts the intestinal microbiota and promotes the overgrowth of Enterobacteriaceae. Cell Host Microbe 2:204. doi:10.1016/j.chom.2007.08.002. 18030708

[3] Barthel M, Hapfelmeier S, Quintanilla-Martínez L, Kremer M, Rohde M, Hogardt M, Pfeffer K, Rüssmann H, Hardt W-D 2003 Pretreatment of mice with streptomycin provides a Salmonella enterica serovar Typhimurium colitis model that allows analysis of both pathogen and host. Infect Immun 71:2839–2858. doi:10.1128/iai.71.5.2839-2858.2003. 12704158 PMC153285

[4] Wilson KH, Silva J, Fekety FR 1981 Suppression of Clostridium difficile by normal hamster cecal flora and prevention of antibiotic-associated cecitis. Infect Immun 34:626–628. 7309245 10.1128/iai.34.2.626-628.1981 PMC350912

[5] Ng KM, Ferreyra JA, Higginbottom SK, Lynch JB, Kashyap PC, Gopinath S, Naidu N, Choudhury B, Weimer BC, Monack DM, Sonnenburg JL 2013 Microbiota-liberated host sugars facilitate post-antibiotic expansion of enteric pathogens. Nature 502:96–99. doi:10.1038/nature12503. 23995682 PMC3825626

[6] Curtis MM, Hu Z, Klimko C, Narayanan S, Deberardinis R, Sperandio V 2014 The gut commensal Bacteroides thetaiotaomicron exacerbates enteric infection through modification of the metabolic landscape. Cell Host Microbe 16:759–769. doi:10.1016/j.chom.2014.11.005. 25498343 PMC4269104

[7] Desai MS, Seekatz AM, Koropatkin NM, Kamada N, Hickey CA, Wolter M, Pudlo NA, Kitamoto S, Terrapon N, Muller A, Young VB, Henrissat B, Wilmes P, Stappenbeck TS, Núñez G, Martens EC 2016 A dietary fiber-deprived gut microbiota degrades the colonic mucus barrier and enhances pathogen susceptibility. Cell 167:1339–1353.e21. doi:10.1016/j.cell.2016.10.043. 27863247 PMC5131798

[8] Zumbrun SD, Melton-Celsa AR, Smith MA, Gilbreath JJ, Merrell DS, O'Brien AD 2013 Dietary choice affects Shiga toxin-producing Escherichia coli (STEC) O157:H7 colonization and disease. Proc Natl Acad Sci U S A 110:E2126–2133. doi:10.1073/pnas.1222014110. 23690602 PMC3677460

[9] Tuttle J, Gomez T, Doyle MP, Wells JG, Zhao T, Tauxe RV, Griffin PM 1999 Lessons from a large outbreak of Escherichia coli O157:H7 infections: insights into the infectious dose and method of widespread contamination of hamburger patties. Epidemiol Infect 122:185–192. doi:10.1017/s0950268898001976. 10355781 PMC2809605

[10] Cameron EA, Sperandio V 2015 Frenemies: signaling and nutritional integration in pathogen-microbiota-host interactions. Cell Host Microbe 18:275–284. doi:10.1016/j.chom.2015.08.007. 26355214 PMC4567707

[11] Kaper JB, Nataro JP, Mobley HL 2004 Pathogenic Escherichia coli. Nat Rev Microbiol 2:123–140. doi:10.1038/nrmicro818. 15040260

[12] Croxen MA, Finlay BB 2010 Molecular mechanisms of Escherichia coli pathogenicity. Nat Rev Microbiol 8:26–38. doi:10.1038/nrmicro2265. 19966814

[13] Carlson-Banning KM, Sperandio V 2018 Enterohemorrhagic Escherichia coli outwits hosts through sensing small molecules. Curr Opin Microbiol 41:83–88. doi:10.1016/j.mib.2017.12.002. 29258058 PMC5862742

[14] Pacheco AR, Curtis MM, Ritchie JM, Munera D, Waldor MK, Moreira CG, Sperandio V 2012 Fucose sensing regulates bacterial intestinal colonization. Nature 492:113–117. doi:10.1038/nature11623. 23160491 PMC3518558

[15] Lebreton F, Willems RJL, Gilmore MS 2014 Enterococcus diversity, origins in nature, and gut colonization *In* Gilmore MS, Clewell DB, Ike Y, Shankar N (ed), Enterococci: from commensals to leading causes of drug resistant infection. Massachusetts Eye and Ear Infirmary, Boston, MA https://www.ncbi.nlm.nih.gov/books/NBK190427/. 24649513

[16] Barnes AMT, Dale JL, Chen Y, Manias DA, Greenwood Quaintance KE, Karau MK, Kashyap PC, Patel R, Wells CL, Dunny GM 2017 Enterococcus faecalis readily colonizes the entire gastrointestinal tract and forms biofilms in a germ-free mouse model. Virulence 8:282–296. doi:10.1080/21505594.2016.1208890. 27562711 PMC5411234

[17] Cameron EA, Curtis MM, Kumar A, Dunny GM, Sperandio V 2018 Microbiota and pathogen proteases modulate type III secretion activity in enterohemorrhagic Escherichia coli. mBio 9:e02204-18. doi:10.1128/mBio.02204-18. 30514785 PMC6282197

[18] Mark Welch JL, Hasegawa Y, McNulty NP, Gordon JI, Borisy GG 2017 Spatial organization of a model 15-member human gut microbiota established in gnotobiotic mice. Proc Natl Acad Sci U S A 114:E9105–E9114. doi:10.1073/pnas.1711596114. 29073107 PMC5664539

[19] Whitaker WR, Shepherd ES, Sonnenburg JL 2017 Tunable expression tools enable single-cell strain distinction in the gut microbiome. Cell 169:538–546.e12. doi:10.1016/j.cell.2017.03.041. 28431251 PMC5576361

[20] Poulsen LK, Lan F, Kristensen CS, Hobolth P, Molin S, Krogfelt KA 1994 Spatial distribution of Escherichia coli in the mouse large intestine inferred from rRNA in situ hybridization. Infect Immun 62:5191–5194. 7927805 10.1128/iai.62.11.5191-5194.1994 PMC303247

[21] Charpentier X, Oswald E 2004 Identification of the secretion and translocation domain of the enteropathogenic and enterohemorrhagic Escherichia coli effector Cif, using TEM-1 beta-lactamase as a new fluorescence-based reporter. J Bacteriol 186:5486–5495. doi:10.1128/JB.186.16.5486-5495.2004. 15292151 PMC490934

[22] DeVinney R, Stein M, Reinscheid D, Abe A, Ruschkowski S, Finlay BB 1999 Enterohemorrhagic Escherichia coli O157:H7 produces Tir, which is translocated to the host cell membrane but is not tyrosine phosphorylated. Infect Immun 67:2389–2398. 10225900 10.1128/iai.67.5.2389-2398.1999 PMC115983

[23] Campellone KG, Robbins D, Leong JM 2004 EspFU is a translocated EHEC effector that interacts with Tir and N-WASP and promotes Nck-independent actin assembly. Dev Cell 7:217–228. doi:10.1016/j.devcel.2004.07.004. 15296718

[24] Kendall MM, Gruber CC, Parker CT, Sperandio V 2012 Ethanolamine controls expression of genes encoding components involved in interkingdom signaling and virulence in enterohemorrhagic Escherichia coli O157:H7. mBio 3:e00050-12. doi:10.1128/mBio.00050-12. 22589288 PMC3372972

[25] Bourgogne A, Garsin DA, Qin X, Singh KV, Sillanpaa J, Yerrapragada S, Ding Y, Dugan-Rocha S, Buhay C, Shen H, Chen G, Williams G, Muzny D, Maadani A, Fox KA, Gioia J, Chen L, Shang Y, Arias CA, Nallapareddy SR, Zhao M, Prakash VP, Chowdhury S, Jiang H, Gibbs RA, Murray BE, Highlander SK, Weinstock GM 2008 Large scale variation in Enterococcus faecalis illustrated by the genome analysis of strain OG1RF. Genome Biol 9:R110. doi:10.1186/gb-2008-9-7-r110. 18611278 PMC2530867

[26] Garmendia J, Frankel G, Crepin VF 2005 Enteropathogenic and enterohemorrhagic Escherichia coli infections: translocation, translocation, translocation. Infect Immun 73:2573–2585. doi:10.1128/IAI.73.5.2573-2585.2005. 15845459 PMC1087358

[27] Guignot J, Segura A, Tran Van Nhieu G 2015 The serine protease EspC from enteropathogenic Escherichia coli regulates pore formation and cytotoxicity mediated by the type III secretion system. PLoS Pathog 11:e1005013. doi:10.1371/journal.ppat.1005013. 26132339 PMC4488501

[28] Hancock LE, Perego M 2004 Systematic inactivation and phenotypic characterization of two-component signal transduction systems of Enterococcus faecalis V583. J Bacteriol 186:7951–7958. doi:10.1128/JB.186.23.7951-7958.2004. 15547267 PMC529088

[29] Kristich CJ, Li YH, Cvitkovitch DG, Dunny GM 2004 Esp-independent biofilm formation by Enterococcus faecalis. J Bacteriol 186:154–163. doi:10.1128/jb.186.1.154-163.2004. 14679235 PMC365672

[30] Thomas VC, Thurlow LR, Boyle D, Hancock LE 2008 Regulation of autolysis-dependent extracellular DNA release by Enterococcus faecalis extracellular proteases influences biofilm development. J Bacteriol 190:5690–5698. doi:10.1128/JB.00314-08. 18556793 PMC2519388

[31] Waters CM, Antiporta MH, Murray BE, Dunny GM 2003 Role of the Enterococcus faecalis GelE protease in determination of cellular chain length, supernatant pheromone levels, and degradation of fibrin and misfolded surface proteins. J Bacteriol 185:3613–3623. doi:10.1128/jb.185.12.3613-3623.2003. 12775699 PMC156229

[32] Thurlow LR, Thomas VC, Narayanan S, Olson S, Fleming SD, Hancock LE 2010 Gelatinase contributes to the pathogenesis of endocarditis caused by Enterococcus faecalis. Infect Immun 78:4936–4943. doi:10.1128/IAI.01118-09. 20713628 PMC2976315

[33] Qin X, Singh KV, Weinstock GM, Murray BE 2001 Characterization of *fsr*, a regulator controlling expression of gelatinase and serine protease in Enterococcus faecalis OG1RF. J Bacteriol 183:3372–3382. doi:10.1128/JB.183.11.3372-3382.2001. 11344145 PMC99635

[34] Qin X, Singh KV, Weinstock GM, Murray BE 2000 Effects of *Enterococcus faecalis fsr* genes on production of gelatinase and a serine protease and virulence. Infect Immun 68:2579–2586. doi:10.1128/iai.68.5.2579-2586.2000. 10768947 PMC97462

[35] Luo W, Donnenberg MS 2006 Analysis of the function of enteropathogenic Escherichia coli EspB by random mutagenesis. Infect Immun 74:810–820. doi:10.1128/IAI.74.2.810-820.2006. 16428723 PMC1360311

[36] Abrami L, Reig N, van der Goot FG 2005 Anthrax toxin: the long and winding road that leads to the kill. Trends Microbiol 13:72–78. doi:10.1016/j.tim.2004.12.004. 15680766

[37] Hughes DT, Clarke MB, Yamamoto K, Rasko DA, Sperandio V 2009 The QseC adrenergic signaling cascade in Enterohemorrhagic E. coli (EHEC). PLoS Pathog 5:e1000553. doi:10.1371/journal.ppat.1000553. 19696934 PMC2726761

[38] Pifer R, Russell RM, Kumar A, Curtis MM, Sperandio V 2018 Redox, amino acid, and fatty acid metabolism intersect with bacterial virulence in the gut. Proc Natl Acad Sci U S A 115:E10712–E10719. doi:10.1073/pnas.1813451115. 30348782 PMC6233112

