# Supplementary figures and images for: Enterococcus faecalis Enhances Expression and Activity of the Enterohemorrhagic Escherichia coli Type III Secretion System
Source: mBio. 2019 Nov 19;10(6):e02547-19. doi: 10.1128/mBio.02547-19 (PMC6867897; doi:10.1128/mBio.02547-19)

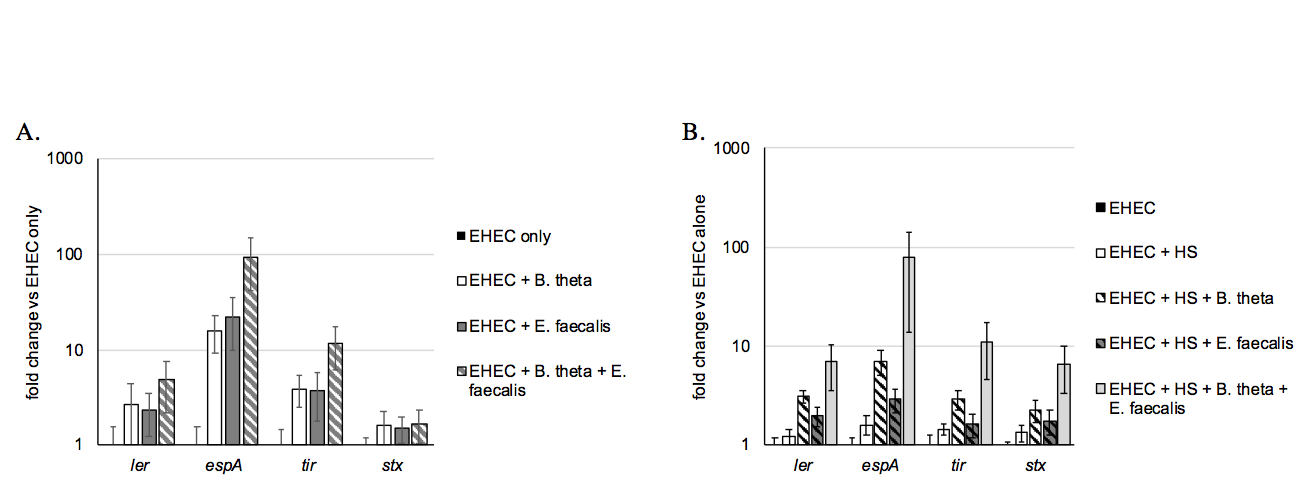

Supplement: FIG S1 [file mBio.02547-19-sf001.tif]

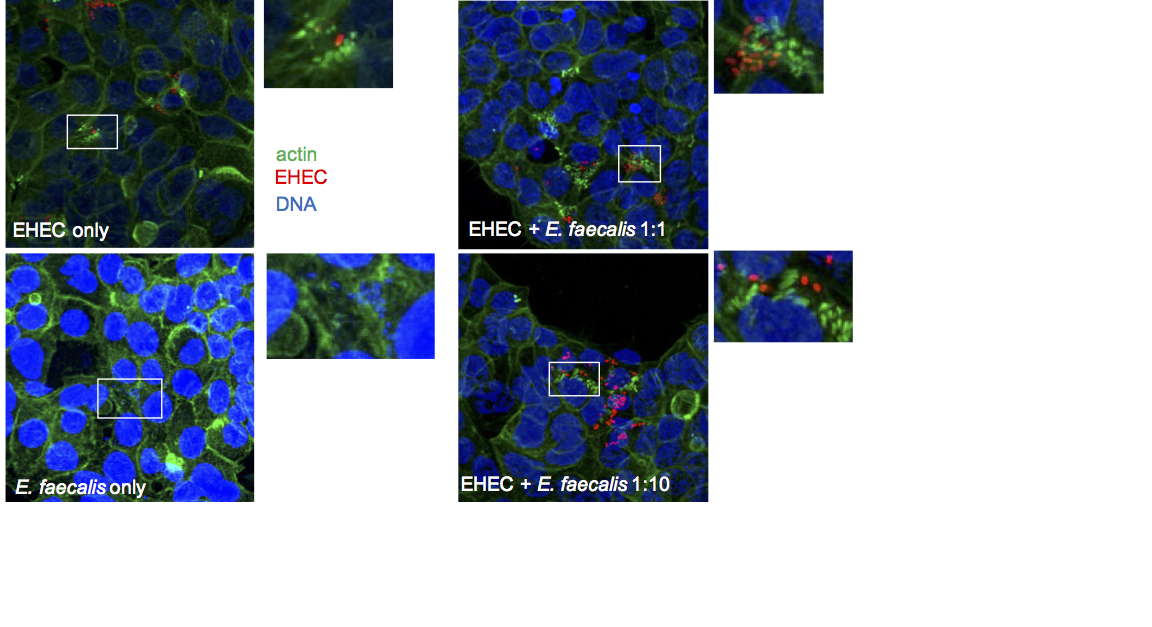

Supplement: FIG S2 [file mBio.02547-19-sf002.tif]

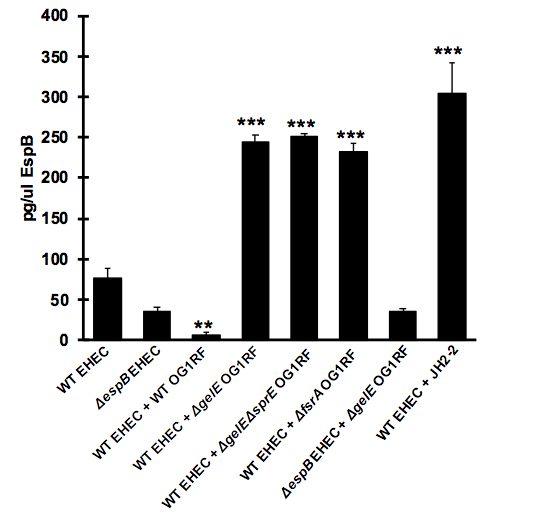

Supplement: FIG S3 [file mBio.02547-19-sf003.tif]

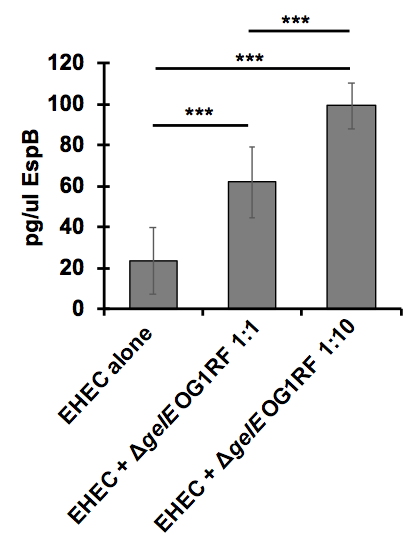

Supplement: FIG S4 [file mBio.02547-19-sf004.tif]

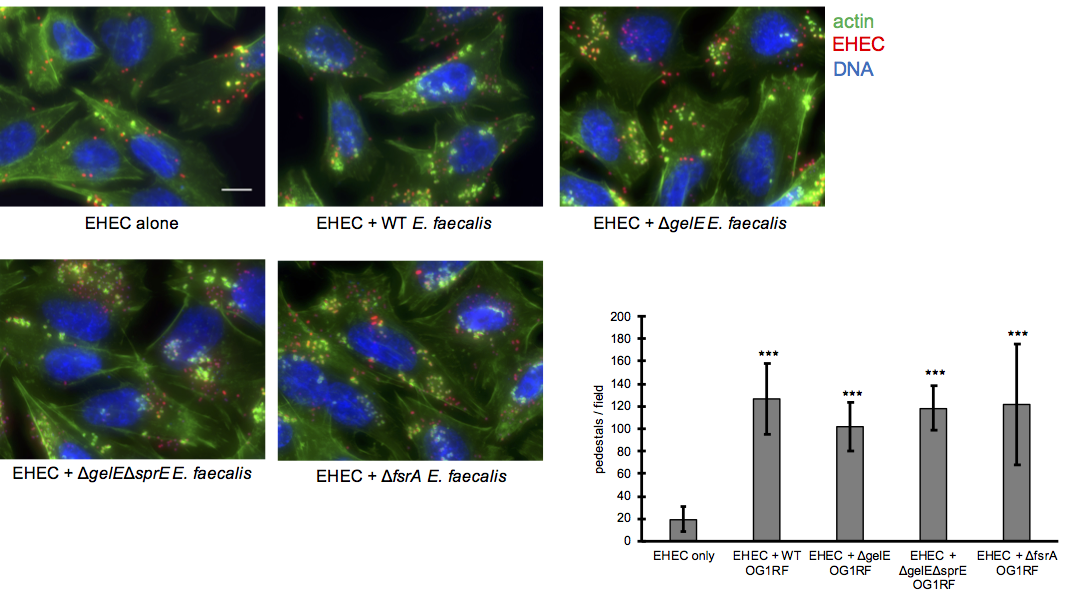

Supplement: FIG S5 [file mBio.02547-19-sf005.tif]

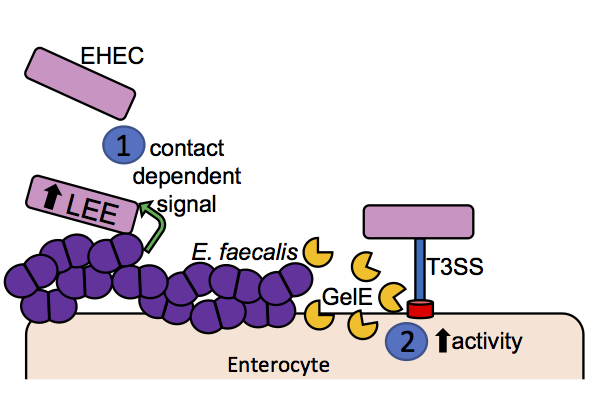

Supplement: FIG S6 [file mBio.02547-19-sf006.tif]
