# Supplementary material for: Enterococcus faecalis Enhances Expression and Activity of the Enterohemorrhagic Escherichia coli Type III Secretion System
Source: mBio. 2019 Nov 19;10(6):e02547-19. doi: 10.1128/mBio.02547-19 (PMC6867897; doi:10.1128/mBio.02547-19)
Supplement: TABLE S1 [file mBio.02547-19-st001.docx]

**Table S1. Strains and Plasmids**

| ***Strains*** | | |
| --- | --- | --- |
| ***Strain*** | ***description*** | ***Reference*** |
| **EHEC** | | |
| Wild-type (WT) | *E. coli* O157:H7 86-24 clinical isolate | (1) |
| Δ*espB* | Isogenic Δ*espB* deletion mutant | (2) |
| *rpsMp_Cm::LacZ* | EHEC strain with engineered unique housekeeping gene. Chloramphenicol resistance gene driven by the *rpsM* promoter and cloned into the *Lac* locus | this work |
| ***Enterococcus*** | | |
| *E. faecalis* V583 | Vancomycin^R^ human blood isolate | (3) |
| *E. faecalis* OG1RF | Rif^R^, Fus^R^ derivative of human isolate OG1 | (4) |
| *E. faecalis* MMH594 | Human blood isolate | (5) |
| *E. faecalis* JH2-2 | Rif^R^, Fus^R^ derivative of clinical isolate JH2 | (6) |
| *E. faecalis* DS5 | Erm^R^, Tet^R^ clinical isolate | (7) |
| *E. faecium* GE-1 | Amp^R^ derivative of clinical isolate | ATCC 51558 |
| *ΔgelE* OG1RF (TX5264) | Isogenic *gelE* deletion mutant | (8) |
| *ΔgelEΔsprE* OG1RF (JRC105) | Isogenic *gelE sprE* deletion mutant | (9) |
| *ΔfsrA* OG1RF (JD100) | Isogenic *fsrA* deletion mutant | (10) |
| **other commensals** | | |
| *Bacteroides thetaiotaomicron* VPI-5482 | Human commensal strain | ATCC 29148 |
| *E. coli* strain HS | Human commensal strain | (11) |
| ***Plasmids*** | | |
| ***Plasmid*** | ***Contents*** | ***reference*** |
| *bla*:pBAD33 | *β-lactamse* under control of pBAD promoter in pBAD33 | (12) |
| *tir-bla*:pBAD33 | *tir-β-lactamse* fusion under control of pBAD promoter in pBAD33 | (12) |
| pDP151 | Constitutive mCherry expression |  |

**Supplementary References**

1. Griffin PM*, et al.* (1988) Illnesses associated with Escherichia coli O157:H7 infections. A broad clinical spectrum. *Ann Intern Med* 109(9):705-712.

2. Carlson-Banning KM & Sperandio V (2016) Catabolite and Oxygen Regulation of Enterohemorrhagic Escherichia coli Virulence. *MBio* 7(6).

3. Sahm DF*, et al.* (1989) In vitro susceptibility studies of vancomycin-resistant Enterococcus faecalis. *Antimicrob Agents Chemother* 33(9):1588-1591.

4. Dunny G, Funk C, & Adsit J (1981) Direct stimulation of the transfer of antibiotic resistance by sex pheromones in Streptococcus faecalis. *Plasmid* 6(3):270-278.

5. Huycke MM, Spiegel CA, & Gilmore MS (1991) Bacteremia caused by hemolytic, high-level gentamicin-resistant Enterococcus faecalis. *Antimicrob Agents Chemother* 35(8):1626-1634.

6. Jacob AE & Hobbs SJ (1974) Conjugal transfer of plasmid-borne multiple antibiotic resistance in Streptococcus faecalis var. zymogenes. *J Bacteriol* 117(2):360-372.

7. Clewell DB, Yagi Y, Dunny GM, & Schultz SK (1974) Characterization of three plasmid deoxyribonucleic acid molecules in a strain of Streptococcus faecalis: identification of a plasmid determining erythromycin resistance. *J Bacteriol* 117(1):283-289.

8. Sifri CD*, et al.* (2002) Virulence effect of Enterococcus faecalis protease genes and the quorum-sensing locus fsr in Caenorhabditis elegans and mice. *Infect Immun* 70(10):5647-5650.

9. Kristich CJ, Chandler JR, & Dunny GM (2007) Development of a host-genotype-independent counterselectable marker and a high-frequency conjugative delivery system and their use in genetic analysis of Enterococcus faecalis. *Plasmid* 57(2):131-144.

10. Dale JL, Cagnazzo J, Phan CQ, Barnes AM, & Dunny GM (2015) Multiple roles for Enterococcus faecalis glycosyltransferases in biofilm-associated antibiotic resistance, cell envelope integrity, and conjugative transfer. *Antimicrob Agents Chemother* 59(7):4094-4105.

11. Levine MM*, et al.* (1978) Escherichia coli strains that cause diarrhoea but do not produce heat-labile or heat-stable enterotoxins and are non-invasive. *Lancet* 1(8074):1119-1122.

12. Cameron EA, Curtis MM, Kumar A, Dunny GM, & Sperandio V (2018) Microbiota and Pathogen Proteases Modulate Type III Secretion Activity in Enterohemorrhagic Escherichia coli. *MBio* 9(6).
