# Supplementary material for: Enterococcus faecalis Enhances Expression and Activity of the Enterohemorrhagic Escherichia coli Type III Secretion System
Source: mBio. 2019 Nov 19;10(6):e02547-19. doi: 10.1128/mBio.02547-19 (PMC6867897; doi:10.1128/mBio.02547-19)
Supplement: TABLE S2 [file mBio.02547-19-st002.docx]

**Table S2. Primers**

| **Primer** | **Sequence 5’**🡪**3’** | **Description** |
| --- | --- | --- |
| *rpoA_*RT_fwd | gcgctcatcttcttccgaat | qPCR |
| *rpoA_*RT_rev | cgcggtcgtggttatgtg | qPCR |
| *ler_*RT_fwd | cgaccaggtctgccc | qPCR |
| *ler_*RT_rev | gcgcggaactcatc | qPCR |
| *espA_*RT_fwd | tcagaatcgcagcctgaaaa | qPCR |
| *espA_*RT_rev | cgaaggatgaggtggttaagct | qPCR |
| *tir_*RT_fwd | gagggagtcaaatagcggtg | qPCR |
| *tir_*RT_rev | atctgaacgaaggctggaag | qPCR |
| *stx_*RT_fwd | accccaccgggcagtt | qPCR |
| *stx_*RT_rev | ggtcaaaacgcgcctgata | qPCR |
| *Cm_*RT_fwd | catcgtaaagaacattttgaggc | qPCR |
| *Cm_*RT_rev | cagctgaacggtctggttatag | qPCR |
| *rpsM*_prom_fwd | ttttttcgcatatttttcttgc | Amplify *rpsM* promoter to clone upstream of Cm |
| *rpsM*_prom_rev_CmSOE | ggtatatccagtgatttttttctccattatgcactcctactatttaatatg | Amplify *rpsM* promoter to clone upstream of Cm |
| Cm_fwd | atggagaaaaaaatcactggatatacc | Amplify Chloramphenicol resistance gene |
| Cm_rev | ttacgccccgccctgccac | Amplify Chloramphenicol resistance gene |
| *rpsM*Cm_LacZ_REDfwd | gcagctggcacbacaggtttcccgactggaaagcgggcagtgagcgcaacttttttcgcatatttttcttgc | insert *rpsM*prom_Cm upstream of LacZ via lambda red recombineering |
| *rpsM*Cm_LacZ_REDrev | cgttgtaaaacgacggccagtgaatctgtaatcatagtcatagctgtatccttacgccccgccctgccac | insert *rpsM*prom_Cm upstream of LacZ via lambda red recombineering |
| *rpsM*Cm_LacZ_checkfwd | gcagctggcacbacaggtttc | amplify *rpsM*prom_Cm_LacZ insertion for sequencing |
| *rpsM*Cm_LacZ_checkrev | cgttgtaaaacgacggccag | amplify *rpsM*prom_Cm_LacZ insertion for sequencing |
